# Dual-Antiplatelet Therapy May Not Be Associated With an Increased Risk of In-hospital Bleeding in Patients With Moderate or Severe Ischemic Stroke

**DOI:** 10.3389/fneur.2021.728111

**Published:** 2021-09-20

**Authors:** Ossama Khazaal, Aaron Rothstein, Muhammad R. Husain, Matthew Broderick, Daniel Cristancho, Sahily Reyes-Esteves, Farhan Khan, Christopher G. Favilla, Steven R. Messé, Michael T. Mullen

**Affiliations:** ^1^Department of Neurology, University of Pennsylvania, Philadelphia, PA, United States; ^2^Department of Neurology, Camden Clark Medical Center/WVU Medicine, Parkersburg, WV, United States

**Keywords:** dual antiplatelet therapy, moderate stroke, severe stroke, secondary prevention, bleeding risk, bleeding rate, hemorrhagic transformation

## Abstract

**Background and Purpose:** Dual antiplatelet therapy (DAPT), compared to single antiplatelet therapy (SAPT), lowers the risk of stroke or death early after TIA and minor ischemic stroke. Prior trials excluded moderate to severe strokes, due to a potential increased risk of bleeding. We aimed to compare in-hospital bleeding rates in SAPT and DAPT patients with moderate or severe stroke (defined by NIHSS ≥4).

**Methods:** We performed a retrospective cohort study of ischemic stroke over a 2-year period with admission NIHSS ≥4. The primary outcome was symptomatic intracranial hemorrhage (ICH) with any change in NIHSS. Secondary outcomes included systemic bleeding and major bleeding, a composite of serious systemic bleeding and symptomatic ICH. We performed analyses stratified by stroke severity (NIHSS 4–7 vs. 8+) and by preceding use of tPA and/or thrombectomy. Univariate followed by multivariate logistic regression evaluated whether DAPT was independently associated with bleeding.

**Results:** Of 377 patients who met our inclusion criteria, 148 received DAPT (39%). Symptomatic ICH was less common with DAPT compared to SAPT (0.7 vs. 6.4%, *p* < 0.01), as was the composite of major bleeding (2.1 vs. 7.6%, *p* = 0.03). Symptomatic ICH was numerically less frequent in the DAPT group, but not statistically significant, when stratified by stroke severity (NIHSS 4–7: 0 vs. 5.9%, *p* = 0.06; NIHSS 8+: 1.5 vs. 6.6%, *p* = 0.18) and by treatment with tPA and/or thrombectomy (Yes: 2.6 vs. 9.1%, *p* = 0.30; No: 0 vs. 2.9%, *p* = 0.25). DAPT was not associated with major bleeding in either the univariate or the multivariate regression.

**Conclusions:** In this single center cohort, symptomatic ICH and the composite of serious systemic bleeding and symptomatic ICH was rare in patients on DAPT. Relative to single antiplatelet therapy DAPT was not associated with an increased risk of in-hospital bleeding in patients with moderate and severe ischemic stroke.

## Introduction

Three randomized controlled trials found that early dual antiplatelet therapy (DAPT), compared to single antiplatelet therapy, lowers the risk of stroke or death after TIA or minor ischemic stroke with only a small absolute increase in the risk of major bleeding (1–3). As a result, guidelines recommend a short course of treatment with DAPT for patients after high risk TIA and minor non-cardioembolic ischemic stroke (4).

Patients with moderate or severe ischemic stroke were excluded from these trials because of a perceived increased risk of hemorrhagic complications. Nonetheless, these patients are at high short-term risk of recurrent ischemic stroke, death, and other major vascular events (5).

Recent studies evaluating the safety of DAPT in moderate to severe ischemic stroke have had limitations. Two studies using a nationwide registry in Korea compared patients with non-minor strokes who were treated with DAPT to those on Aspirin alone. They found that hemorrhagic stroke rates did not significantly differ between groups, and that recurrent vascular events were lower in the DAPT group (6, 7). These studies did not report details about the type of hemorrhagic transformation, did not report systemic bleeding, and were conducted in a homogenous patient population which might limit generalizability.

With this background, we completed a single center retrospective cohort comparing the safety of single and dual antiplatelet therapy in moderate and severe ischemic stroke, defined by a National Institute of Health Stroke Scale (NIHSS) of 4 or greater. We hypothesized that bleeding rates in patients on DAPT would be similar to patients on single antiplatelet therapy.

## Methods

This was a single center retrospective cohort study. After institutional review board approval, we searched our hospital's stroke registry to identify all patients admitted between January 1st 2017 and November 1st 2019 with ischemic stroke who were age >18 years, and who had an admission NIHSS ≥4. Patients who received at least one dose of any form of therapeutic anticoagulation during their hospitalization were excluded.

We collected demographic characteristics, vascular risk factors, NIHSS, stroke mechanism, antiplatelet treatments throughout admission, and bleeding outcomes. Given the retrospective nature of this analysis, there was no pre-specified criteria for the use of DAPT. The primary outcome was symptomatic intracranial hemorrhage (sICH) with any change in NIHSS. Secondary outcomes included asymptomatic ICH, sICH with a change in NIHSS of 4 or more points, ICH classified according to the Heidelberg criteria (8), any systemic bleeding, and major bleeding (sICH or systemic bleeding that required transfusion, pressors, escalation of care, or causing death) (9). For each bleeding event it was determined if the bleeding occurred before or after initiation of DAPT. Only bleeding that occurred after initiation of DAPT was attributed to DAPT.

Variables were summarized by means and standard deviations or medians and interquartile range for continuous variables and frequencies/proportions for categorical variables. Differences between groups were evaluated using chi-squared tests for categorical variables and parametric (*t*-test) or non-parametric tests (Kruskal Wallis, Wilcoxon rank sum) as appropriate. We evaluated for differences in bleeding outcomes among patients on DAPT and those not on DAPT. We also performed secondary analysis stratified by NIHSS (4–7 vs. 8+) and based on whether patients received tPA and/or thrombectomy. Finally, multivariate logistic regression was used to determine if DAPT was independently associated with bleeding. Age, NIHSS, and variables significant in univariate analysis with *p* < 0.10 were included. Analysis was performed using Stata 15.0 (StataCorp LP. College Station, TX, 2017).

## Results

A total of 377 patients met our inclusion criteria. Patients were divided into two groups based on the antiplatelet treatment they received throughout their admission. There were 148 patients who were treated with DAPT at any time during the hospitalization and 229 who were not (single antiplatelet therapy). All the DAPT subjects were treated with the combination of aspirin and clopidogrel. Of these 148 patients, 28.4% were loaded with 600 mg of clopidogrel and then maintained on 75 mg daily, 20.3% were loaded with 300 mg and then maintained on 75 mg daily, and 50.7% were started on 75 mg of clopidogrel daily without receiving a loading dose. Baseline demographics are summarized in Table 1. Patients in the DAPT group were more likely to have traditional vascular risk factors and large artery atherosclerosis as the mechanism of stroke. The median admission duration was 5 days and the median time from admission to start of DAPT was 1 day (IQR 0–2). About half (49%) of the patients in the DAPT group were loaded with clopidogrel (either 300 mg or 600 mg).

**Table 1:** Baseline characteristics.

	**SAPT (*n* = 229)**	**DAPT (*n* = 148)**	***P*-value**
Age, mean	66.4	67.3	0.58
Sex, % female	53.3	44.6	0.10
**Medical history (%)**			
Hypertension	73.4	86.5	<0.01
Hyperlipidemia	50.7	62.8	0.02
Diabetes mellitus	32.8	43.9	0.03
Coronary artery disease	15.7	27.0	<0.01
Active smoking	25.8	28.4	0.58
Chronic kidney disease	13.1	17.6	0.23
Congestive heart failure	10.5	12.2	0.61
Atrial fibrillation	11.8	3.4	<0.01
**Antiplatelet prior to admission (%)**			
None	61.6	38.5	<0.01
SAPT	35.8	39.9	0.43
DAPT	2.6	21.6	<0.01
Admission NIHSS, median (IQR)	9 (6–17)	7 (5–11)	<0.01
**Acute stroke treatments (%)**			
tPA	38.9	15.5	<0.01
Thrombectomy	29.3	19.6	0.04
**Stroke mechanism (%)**			
Large artery atherosclerosis	11.8	46.0	<0.01
Small vessel disease	9.2	8.8	0.90
Cardioembolic	22.7	6.1	<0.01
Cryptogenic	39.3	17.6	<0.01
>1 mechanism	7.0	10.1	0.28
Other	10.0	11.5	0.66
Length of stay, median (IQR)	5 (3–10)	5 (3–9.5)	0.63
**Disposition (%)**			
Home	28.8	23.0	0.21
Acute rehab	56.8	71.0	<0.01
Skilled nursing facility	9.2	3.4	0.03

*SAPT, single antiplatelet therapy; DAPT, dual antiplatelet therapy; tPA, tissue plasminogen activator*.

Bleeding outcomes are shown in Table 2. Of the 148 patients that received DAPT during their hospitalization, 7 had ICH before DAPT was initiated. For this analysis these 7 subjects are included in the “Not DAPT” group, as their bleeding event occurred while on single or no antiplatelet therapy. Asymptomatic, sICH with any change in NIHSS, and sICH with NIHSS change ≥4 were all less common in the DAPT group. Only 1 of 141 subjects in the DAPT group had a sICH with NIHSS change ≥4. This patient had a PH2 hemorrhagic transformation which occurred on the same day that the patient was loaded with 600 mg of clopidogrel. There were 15 subjects in the Not DAPT group with symptomatic ICH, of which 12 (80%) occurred within 1 day of hospital admission. The composite outcome of major bleeding was seen in 2.1% of subjects on DAPT and 7.6% of patients not on DAPT (*p* = 0.03).

**Table 2:** Bleeding rates by antiplatelet therapy at the time of event.

	**Not DAPT (*N* = 236)**	**DAPT (*N* = 141)**	***P*-value**
Any systemic bleeding	3.8%	5.0%	0.59
Gastrointestinal	1.3%	0.7%	1.00
Genitourinary	0.8%	2.1%	0.37
Other	1.7%	2.1%	0.72
Serious systemic bleeding	1.3%	1.4%	1.00
Major bleeding	7.6%	2.1%	0.03
Asymptomatic ICH	11.0%	1.4%	<0.01
Symptomatic ICH (NIHSS any change)	6.4%	0.7%	<0.01
Symptomatic ICH (NIHSS change >/=4)	1.3%	0.7%	1.00
**ICH classification**			
HI1	5.1%	0.7%	0.04
HI2	3.4%	0%	0.03
PH1	2.1%	0%	0.16
PH2	4.2%	0.7%	0.06
Other (Any SAH, IVH, SDH)	8.9%	1.4%	<0.01

*DAPT, dual antiplatelet therapy; ICH, intracranial hemorrhage; NIHSS, National Institute of Health stroke scale; HI1, hemorrhagic transformation type 1 (defined as small petechiae without mass effect); HI2, hemorrhagic transformation type 2 (defined as more confluent petechiae without mass effect); PH1, parenchymal hematoma type 1 (defined as hematoma <30% of infarct bed with some mild mass effect); PH2, parenchymal hematoma type 2 (defined as hematoma >30% of infarct bed with significant mass effect); SAH, subarachnoid hemorrhage; IVH, intraventricular hemorrhage; SDH: subdural hemorrhage*.

In the stratified analysis (Tables 3, 4), sICH was numerically less common in the DAPT group, but the differences were not significant (NIHSS 4–7: 0 vs. 5.9%, *p* = 0.06; NIHSS 8+: 1.5 vs. 6.6%, *p* = 0.18). Similarly, when stratified by treatment with tPA and/or thrombectomy, sICH was less frequent in the DAPT group but the differences were not significant (Yes: 2.6 vs. 9.1%, *p* = 0.30; No: 0 vs. 2.9%, *p* = 0.25). Of the 13 sICH that occurred in patients treated with tPA or thrombectomy 10 (77%) occurred within 1 day of admission.

**Table 3:** Bleeding rates among lower vs. higher NIHSS groups subcategorized by antiplatelet therapy groups.

**NIHSS**	**4–7 (** ***n*** **=** **160)**			**≥8 (** ***n*** **=** **217)**		
	**Not DAPT (*n* = 85)**	**DAPT (*n* = 75)**	***P*-value**	**Not DAPT (*n* = 151)**	**DAPT (*n* = 66)**	***P*-value**
Major bleeding	5.9%	0%	0.06	8.6%	4.6%	0.40
Asymptomatic ICH	5.9%	2.7%	0.45	13.9%	0%	<0.01
Symptomatic ICH (NIHSS any change)	5.9%	0%	0.06	6.6%	1.5%	0.18
Symptomatic ICH (NIHSS change >/=4)	1.2%	0%	1.00	1.3%	1.5%	1.00
**ICH classification**						
HI1	3.5%	1.3%	0.62	6.0%	0%	0.06
HI2	3.5%	0%	0.25	3.3%	0%	0.33
PH1	0%	0%	1.00	3.3%	0%	0.33
PH2	2.4%	0%	0.50	5.3%	1.5%	0.28
Other (Any SAH, IVH or SDH)	4.7%	1.3%	0.37	11.3%	1.5%	0.02

*DAPT, dual antiplatelet therapy; ICH, intracranial hemorrhage; NIHSS, National Institute of Health stroke scale; HI1, hemorrhagic transformation type 1 (defined as small petechiae without mass effect); HI2, hemorrhagic transformation type 2 (defined as more confluent petechiae without mass effect); PH1, parenchymal hematoma type 1 (defined as hematoma <30% of infarct bed with some mild mass effect); PH2, parenchymal hematoma type 2 (defined as hematoma >30% of infarct bed with significant mass effect); SAH, subarachnoid hemorrhage; IVH, intraventricular hemorrhage; SDH, subdural hemorrhage*.

**Table 4:** Bleeding rates among those who received tPA or thrombectomy and those who didn't subcategorized by antiplatelet therapy groups.

**Received tPA or Thrombectomy**	**Yes (** ***n*** **=** **171)**			**No (** ***n*** **=** **206)**		
	**Not DAPT (*n* = 132)**	**DAPT (*n* = 39)**	***P*-value**	**Not DAPT (*n* = 104)**	**DAPT (*n* = 102)**	***P*-value**
Major bleeding	10.6%	2.6%	0.20	3.9%	2%	0.68
Asymptomatic ICH	15.2%	2.6%	0.05	5.8%	1.0%	0.12
Symptomatic ICH (NIHSS any change)	9.1%	2.6%	0.30	2.9%	0%	0.25
Symptomatic ICH (NIHSS change >/=4)	2.3%	2.6%	1.00	0%	0%	1.00
**ICH classification**						
HI1	3.8%	2.6%	1.00	6.7%	0%	0.01
HI2	5.3%	0%	0.35	1.0%	0%	1.00
PH1	3.8%	0%	0.59	0%	0%	1.00
PH2	6.8%	2.6%	0.46	1.0%	0%	1.00
Other (Any SAH, IVH or SDH)	13.6%	2.6%	0.08	2.9%	1.0%	0.62

*tPA, tissue plasminogen activator; DAPT, dual antiplatelet therapy; ICH, intracranial hemorrhage; NIHSS, National Institute of Health stroke scale; HI1, hemorrhagic transformation type 1 (defined as small petechiae without mass effect); HI2, hemorrhagic transformation type 2 (defined as more confluent petechiae without mass effect); PH1, parenchymal hematoma type 1 (defined as hematoma <30% of infarct bed with some mild mass effect); PH2, parenchymal hematoma type 2 (defined as hematoma >30% of infarct bed with significant mass effect); SAH, subarachnoid hemorrhage; IVH, intraventricular hemorrhage; SDH, subdural hemorrhage*.

DAPT was not associated with major bleeding in either the univariate (Table 5) or the multivariate regression analysis (Table 6).

**Table 5:** Univariate analysis.

**Variable**	**OR**	**95% CI**	***P*-value**
**Age**			
<60	Ref		
60–79	1.70	0.53–5.48	0.37
80+	2.30	0.63–8.45	0.21
Male gender	0.59	0.24–1.47	0.26
**Stroke etiology**			
Large vessel disease	Ref		
Small vessel disease	No bleeding events		
Cardioembolic	2.72		0.09
Other	0.46	0.85–8.73	0.49
More than 1 mechanism	1.24	0.05–4.08	0.80
Cryptogenic	0.81	0.23–2.89	0.745
Cardioembolic etiology	3.52	1.39–8.90	<0.01
**NIHSS**			
4–7	Ref		
8+	2.47	0.88–6.88	0.08
**Past medical history**			
Hypertension	1.68	0.48–5.86	0.413
Hyperlipidemia	1.65	0.65–4.19	0.29
Diabetes mellitus	1.04	0.42–2.59	0.93
Coronary artery disease	1.25	0.44–3.54	0.67
Chronic kidney disease	1.87	0.66–5.32	0.24
Congestive heart failure	1.35	0.38–4.81	0.64
Atrial fibrillation	6.62	2.45–17.89	<0.01
End stage liver disease	7.39	1.35–40.59	0.02
Prior GI Bleeding	1.75	0.49–6.26	0.39
Prior GU Bleeding	1.57	0.19–12.76	0.67
Prior hemorrhagic transformation	9.09	3.09–26.74	<0.01
Current smoking	1.39	0.55–3.56	0.49
tPA or thrombectomy	3.21	1.22–8.45	0.02

*Ref, Reference; tPA, tissue plasminogen activator; GI, gastrointestinal; GU, genitourinary*.

**Table 6:** Multivariate analysis for variables associated with Major Bleeding.

**Variable**	**OR**	**95% CI**	***P*-value**
**Age**			
<60	Ref		
60–79	1.42	0.41–4.89	0.58
80+	1.44	0.31–6.71	0.64
Male gender	0.73	0.26–2.04	0.55
Cardioembolic etiology	1.36	0.36–5.09	0.65
**NIHSS**			
4–7	Ref		
8+	1.84	0.59–5.76	0.30
**Past medical history**			
Atrial fibrillation	1.99	0.41–9.62	0.39
End stage liver disease	6.73	0.63–71.78	0.12
Prior hemorrhagic transformation	2.95	0.76–11.50	0.12
tPA or thrombectomy	1.63	0.56–4.72	0.37
DAPT at time of bleed	0.34	0.08–1.50	0.15

*Ref, Reference; tPA, tissue plasminogen activator; DAPT, dual antiplatelet therapy*.

## Discussion

Early treatment with DAPT has been shown to be beneficial in high-risk TIA and minor ischemic stroke, although the benefit in moderate to severe stroke is uncertain. In this single center retrospective study, we found that patients with non-minor stroke defined by NIHSS ≥4 who were treated with DAPT did not have higher rates of major bleeding during their hospitalization than those who were not treated with DAPT.

Both asymptomatic and sICH were less common in the DAPT group than the group not on DAPT. We observed relatively high rates of ICH in the single antiplatelet therapy group. This outcome was biologically unexpected and we believe it reflects, in part, careful screening for asymptomatic ICH at our institution. It is probable that clinicians were less likely to use dual antiplatelet therapy in patients with asymptomatic ICH and this selection bias may have contributed to the observed difference in asymptomatic ICH between groups. Importantly, Table 4 shows that 77% of asymptomatic ICH and 80% of sICH in the single antiplatelet group occurred in patients treated with tPA and/or thrombectomy. These hemorrhages likely reflect complications of those treatments and further biased the results toward a higher overall ICH rate in the single antiplatelet therapy group. While this bias impacts the comparison between groups in this study, it is still reassuring that ICH in the DAPT group was very low. Only 1 of the subjects had sICH after initiation of DAPT, and there was with no signal of increased ICH risk in the DAPT group in the stratified analyses. A prospective, randomized trial is needed to determine if DAPT is effective in moderate and severe stroke. Such a trial would likely exclude patients treated with tPA and/or thrombectomy, particularly if those treatments were complicated by ICH, and our data suggests that in such a population treatment with DAPT is associated with very low rates of sICH.

Major bleeding was seen in 2.1% of patients treated with DAPT. This is numerically higher than the major bleeding rates reported in the CHANCE (0.2%), POINT (0.9%), and THALES (0.5%) trials (1–3), but similar to the major bleeding rate reported in the medical arm of the SAMMPRIS Trial (2.2%) (10). It may be that the overall higher rates of bleeding in our study reflect a higher risk in this population, compared to a TIA and minor stroke population, rather than an effect specific to DAPT.

As a retrospective cohort, this study has limitations. The most important is the potential for selection bias. As discussed above, clinicians likely selected patients for DAPT who they felt were at a lower, or at least acceptable, risk of ICH, biasing the results toward a higher overall hemorrhage rate in the single antiplatelet group. We did not record the rationale for using DAPT. Additionally, only about half of the patients in this study were loaded with clopidogrel, which may also have contributed to lower bleeding rates in the DAPT group. We defined non-minor stroke using NIHSS, rather than infarct volume. This definition is consistent with published randomized trials which used NIHSS to define minor stroke. NIHSS is correlated with infarct volume (11) but nonetheless, direct volume measurements would have a stronger correlation with bleeding risk (12). Outcomes were defined as bleeding during hospitalization. Median time from admission to start of DAPT was 1 day, with an average length of stay of 5 days. Hemorrhagic transformation risk is likely highest early after ischemic stroke, but it is possible that there were bleeding events after discharge that were missed. Finally, the relatively small number of outcome events limited the statistical power.

## Conclusion

In this single center retrospective study, we found that DAPT was associated with a very low rate of sICH in patients with moderate and severe ischemic stroke defined by NIHSS ≥4. A randomized trial is needed to confirm this finding and to determine if DAPT is effective at preventing recurrent stroke in this population.

## Data Availability Statement

The raw data supporting the conclusions of this article will be made available by the authors upon request.

## Ethics Statement

The studies involving human participants were reviewed and approved by Penn IRB. The Ethics Committee waived the requirement of written informed consent for participation.

## Author Contributions

OK contributed to the writing of the paper. AR, MH, MB, DC, SR-E, FK, CF, SM, and MM contributed to data collection and review of written portion. MM contributed to the statistical analysis and writing of the paper as well as being the senior author. All authors contributed to the article and approved the submitted version.

## Conflict of Interest

The authors declare that the research was conducted in the absence of any commercial or financial relationships that could be construed as a potential conflict of interest.

## Publisher's Note

All claims expressed in this article are solely those of the authors and do not necessarily represent those of their affiliated organizations, or those of the publisher, the editors and the reviewers. Any product that may be evaluated in this article, or claim that may be made by its manufacturer, is not guaranteed or endorsed by the publisher.

## Abbreviations

DAPT: Dual antiplatelet therapy
SAPT: Single antiplatelet therapy
sICH: Symptomatic intracranial hemorrhage.
